# Self efficacy for fruit, vegetable and water intakes: Expanded and abbreviated scales from item response modeling analyses

**DOI:** 10.1186/1479-5868-7-25

**Published:** 2010-03-29

**Authors:** Tom Baranowski, Kathleen B Watson, Christine Bachman, Janice C Baranowski, Karen W Cullen, Debbe Thompson, Anna-Maria Siega Riz

**Affiliations:** 1 Children's Nutrition Research Center, Department of Pediatrics, Baylor College of Medicine, 1100 Bates Street, Houston, Texas 77030-2600, USA; 2 University of Houston, Downtown Campus, Houston, TX, USA; 3 University of North Carolina, Chapell Hill, NC, USA

## Abstract

**Objective:**

To improve an existing measure of fruit and vegetable intake self efficacy by including items that varied on levels of difficulty, and testing a corresponding measure of water intake self efficacy.

**Design:**

Cross sectional assessment. Items were modified to have easy, moderate and difficult levels of self efficacy. Classical test theory and item response modeling were applied.

**Setting:**

One middle school at each of seven participating sites (Houston TX, Irvine CA, Philadelphia PA, Pittsburg PA, Portland OR, rural NC, and San Antonio TX).

**Subjects:**

714 6^th ^grade students.

**Results:**

Adding items to reflect level (low, medium, high) of self efficacy for fruit and vegetable intake achieved scale reliability and validity comparable to existing scales, but the distribution of items across the latent variable did not improve. Selecting items from among clusters of items at similar levels of difficulty along the latent variable resulted in an abbreviated scale with psychometric characteristics comparable to the full scale, except for reliability.

**Conclusions:**

The abbreviated scale can reduce participant burden. Additional research is necessary to generate items that better distribute across the latent variable. Additional items may need to tap confidence in overcoming more diverse barriers to dietary intake.

## Introduction

Fruit and vegetable (FV) consumption among children is generally considered health promoting. Self efficacy (SE), a person's confidence in being able to perform a behavior (e.g. eat FV), originated in Bandura's Social Cognitive Theory [1] and has been incorporated into several theories [2-4] predicting behavior. Inconsistencies exist across studies with children as to whether SE was related to FV intake [5,6], and when detected, the relationships were low [2,7,8]. There has been concern for high response burden [2], however, when only one or two SE items were used, the expected relationships were not detected [2]. Characteristics of the existing full scale may account for the inconsistencies and low correlations. Although classical test theory reliability was acceptable for the original scale (alpha = 0.88) [9], item response modeling (IRM), a psychometric procedure that fits a latent variable to items, was also used to enhance understanding of the FV SE scale. The latent variable reflects the participants' difficulty of agreeing with an item. The items should be distributed across the full range of the distribution of participants along the difficulty of response scale (from the easiest to the most difficult to agree with). However the IRM analyses revealed that the items did not adequately represent either end of the distribution [10], thereby limiting content validity. Although the items in the available scale assessed the generality of SE (e.g., at alternative specific meals and snacks) [9], they did not tap the levels of SE: easy, moderate and difficult forms of the behavior as specified by Social Cognitive Theory [11]. We hypothesized that generating items at different levels of difficulty of the behavior would improve the distributional characteristics of the items across the difficulty to agree with latent variable.

## Experimental Methods

Starting with the original scale, this paper 1) generated FV SE items to assess levels [11], 2) applied IRM to the FV SE items, and 3) reduced the number of items by selecting a subset of non-redundant items. Parallel analyses were conducted on a new scale of drinking water (W) SE.

### Design

Data were collected as formative research performed for the Studies to Treat or Prevent Pediatric Type 2 Diabetes (STOPP-T2D) - Prevention [12]. STOPP-T2D - Prevention was a multi-site study designed to reduce the risk factors for type 2 diabetes among middle school children [13]. Data were collected at seven field centers (Baylor College of Medicine, Houston TX; University of California Irvine, CA; University of North Carolina at Chapel Hill, NC; Oregon Health & Sciences University, Portland OR; University of Pittsburgh, PA; University of Pennsylvania, Philadelphia PA; and University of Texas Health Science Center - San Antonio, TX). The study was coordinated by the Biostatistics Center at George Washington University (Rockville MD). Approval was obtained from the Institutional Review Boards at each field center and the coordinating center, and written informed parent consent and child assent were obtained for all participants.

### Sample

Participants were 942 6^th ^grade students recruited from seven middle schools, one from each of the seven sites. Schools were required to have at least 40% of students from an ethnic group at increased risk of type 2 diabetes mellitus (African American, Native American or Hispanic). This was a convenience sample of schools. A comprehensive recruitment approach was used including presentations to students in assemblies and classrooms. Volunteering 6^th ^grade students were asked to bring consent and assent forms home for parent signatures. This was a self selected sample of students within schools.

### FV SE Scale Enhancement

To spread the FV SE scale items across the latent variable, the previous scale's 24 items which were generated from qualitative research [10] were expanded to 43 items. Theory based procedures [11] specified generating easy, moderate and difficult versions of each behavior by varying the number of portions at a meal or snack, and the frequency of a behavior in the week, across the various meals, locations and situations included in the original set of items. For example: "eat 1 portion of fruit for a snack at home at least one time" (item 181) and "eat 1 portion of fruit for a snack at home at least 4 days a week" (item 182). The coauthors constituted a multidisciplinary expert panel. Several iterations of this review with revisions were conducted until all were satisfied with the items. Each item asked "How sure are you that you can ...." (Table 1). Dichotomous "sure" and "not sure" response categories were selected because previous work in this age group suggested that participant responses usually fell within these two categories [10]. Cognitive interviewing was conducted on these items with 10 middle school students of diverse gender and ethnicity at the Houston site to ensure that target aged children understood the items and response scale. Minor changes were made in wording. Similar procedures were used to generate water (W) SE items.

**Table 1:** Results from Classical Test Theory and Item Response Modeling Analyses of the Fruit Self-Efficacy Scales

Scale/Item			M (SD)	CITC	Factor	Est (SE)	Infit
Item stem for **Fruit (N = 664)**: How sure are you that you can...							

181	×	eat 1 portion of fruit for a snack at home at least one time?	0.82 (0.38)	0.47	0.70	-0.94 (0.07)	0.94
169		drink a glass of 100% juice or eat a piece of fruit at breakfast at least one time?	0.78 (0.40)	0.42	0.63	-0.64 (0.07)	0.97
173	×	eat 1 portion of fruit at lunch at least one time on a school day?	0.78 (0.41)	0.43	0.64	-0.63 (0.07)	1.00
197		eat 1 portion of fruit at a cafeteria place at least one time?	0.77 (0.41)	0.49	0.70	-0.56 (0.07)	0.92
165		ask someone in family to buy your favorite fruit or vegetable at least one time?	0.77 (0.41)	0.43	0.63	-0.56 (0.07)	1.02
187	×	eat 1 portion of fruit for dinner or supper at home at least one time?	0.77 (0.42)	0.43	0.63	-0.49 (0.07)	1.00
177	×	eat 1 portion of fruit for lunch at least one time on a non-school day, including weekend?	0.75 (0.43)	0.41	0.60	-0.39 (0.07)	1.01
185		ask someone in family to serve 1 fruit instead of usual dessert for dinner at least 1 time?	0.75 (0.43)	0.48	0.68	-0.36 (0.07)	0.95
182	×	eat 1 portion of fruit for a snack at home at least 4 days a week?	0.72 (0.44)	0.48	0.68	-0.22 (0.07)	0.93
174		eat 1 portion of fruit at lunch most school days?	0.71 (0.45)	0.45	0.65	-0.11 (0.07)	1.01
178	×	eat 1 portion of fruit for lunch most non-school days, including weekends?	0.69 (0.46)	0.47	0.66	-0.02 (0.07)	0.93
198		eat 1 portion of fruit most times when you eat at a cafeteria?	0.68 (0.46)	0.42	0.60	0.04 (0.32)	1.03
193	×	eat 1 portion of fruit at a fast food place at least one time?	0.67 (0.47)	0.39	0.54	0.15 (0.07)	1.04
170		drink a glass of 100% juice or eat a piece of fruit at breakfast at least 4 days a week?	0.66 (0.47)	0.47	0.66	0.17 (0.07)	0.97
167	×	ask someone in family to buy 3 fruit or vegetables at least one time?	0.66 (0.47)	0.33	0.47	0.21 (0.07)	1.12
189		eat 2 portions of fruit at least 4 days a week?	0.65 (0.48)	0.50	0.69	0.27 (0.07)	0.95
186	×	ask someone in family to serve 1 fruit instead of your usual dessert/dinner most nights?	0.64 (0.48)	0.46	0.64	0.31 (0.07)	0.98
188		eat 1 portion of fruit for dinner or supper at home most nights?	0.61 (0.48)	0.42	0.57	0.49 (0.07)	1.03
166	×	ask someone in family to buy your favorite fruit or vegetable every week?	0.59 (0.49)	0.42	0.58	0.57 (0.07)	1.06
190		eat 2 portions of fruit at least 4 days a week, even when you are stressed?	0.57 (0.49)	0.46	0.64	0.70 (0.07)	0.95
168	×	ask someone in family to buy 3 fruit or vegetables every week?	0.54 (0.49)	0.39	0.54	0.90 (0.07)	1.09
194	×	eat 1 portion of fruit most times when you eat at a fast food place?	0.50 (0.50)	0.36	0.49	1.11 (0.07)	1.11
Item stem for **Vegetables (n = 659)**: How sure are you that you can...							
183	×	ask someone in family to serve 2 vegetables for dinner at least one time?	0.71 (0.45)	0.44	0.66	-0.79 (0.07)	1.01
199		eat 1 portion of vegetables at a cafeteria at least one time?	0.69 (0.46)	0.50	0.72	-0.70 (0.07)	0.92
179	×	cut up 1 portion of vegetables and eat it with a dip for a snack at least one time?	0.67 (0.47)	0.40	0.58	-0.58 (0.07)	1.00
175		eat 1 portion of vegetables for lunch at least 1 time on non-school day, including weekend?	0.65 (0.47)	0.39	0.58	-0.46 (0.07)	1.12
171	×	eat 1 portion of vegetables at lunch at least one time on a school day?	0.63 (0.48)	0.43	0.63	-0.29 (0.07)	1.07
195		eat 1 portion of vegetables at a fast food place at least one time?	0.61 (0.48)	0.45	0.65	-0.18 (0.07)	1.05
184	×	ask someone in family to serve 2 vegetables for dinner most nights?	0.55 (0.50)	0.56	0.77	0.14 (0.07)	0.91
180		cut up 1 portion of vegetables and eat it with a dip for a snack at least 4 days a week?	0.55 (0.50)	0.39	0.56	0.15 (0.07)	1.09
176		eat 1 portion of vegetables for lunch for most non-school days, including weekends?	0.55 (0.50)	0.52	0.72	0.15 (0.07)	0.95
200	×	eat 1 portion of vegetables most times when you eat at a cafeteria?	0.52 (0.49)	0.54	0.75	0.36 (0.24)	0.97
172		eat 1 portion of vegetables at lunch at least 4 days a week at school?	0.50 (0.50)	0.53	0.73	0.44 (0.07)	0.98
191	×	eat 3 portions of vegetables at least 4 days a week?	0.50 (0.50)	0.56	0.76	0.47 (0.07)	0.91
196	×	eat 1 portion of vegetables most times when you eat at a fast food place?	0.49 (0.50)	0.50	0.70	0.49 (0.07)	1.05
192	×	eat 3 portions of vegetables at least 4 days a week, even when you are stressed?	0.44 (0.49)	0.55	0.76	0.81 (0.07)	0.95
Item stem for **Water (n = 640)**: How sure are you that you can...							
205	×	drink only water whenever you are thirsty for at least one day?	0.79 (0.40)	0.30	0.51	-0.78 (0.07)	1.10
202		drink 4 glasses or bottles of water at least 4 days a week?	0.77 (0.41)	0.50	0.82	-0.59 (0.07)	0.93
201	×	drink 4 glasses or bottles of water at least one day?	0.75 (0.43)	0.42	0.73	-0.43 (0.07)	0.94
206	×	drink only water whenever you are thirsty at least 4 days a week?	0.71 (0.45)	0.31	0.52	-0.13 (0.07)	1.10
204	×	drink 6 glasses or bottles of water at least 4 days a week?	0.60 (0.49)	0.45	0.75	0.57 (0.07)	0.96
207		drink 6 glasses or bottles of water at least 4 days a week, even when stressed?	0.59 (0.49)	0.44	0.71	0.63 (0.17)	0.99
203	×	drink 6 glasses or bottles of water at least one day?	0.57 (0.49)	0.47	0.78	0.74 (0.07)	0.98

% Variance Explained for Fruit (Factor 1/Factor 2) (38.8%/7.5%)-Cronbach's alpha/Person-separation reliability (full scale = 0.86/0.82; reduced scale = 0.75/0.74)

% Variance Explained for Vegetables (Factor 1/Factor 2) (47.1%/9.5%)-Cronbach's alpha/Person-separation reliability (full scale = 0.84/0.83; reduced scale = 0.70/0.76)

% Variance Explained for Water (Factor 1/Factor 2) (48.7%/20.3%)-Cronbach's alpha/Person-separation reliability (full scale = 0.77/0.66; reduced scale = 0.55/0.61)

Mean (M); standard deviation (SD), corrected item-total correlation (CITC); item response modeling item difficulty estimate and standard error [Est (SE)], Infit statistic (acceptable range 0.75-1.33); Self efficacy item scale: not sure (0), sure (1); "x" identifies items used in the reduced scales

### Measures Data Collection Procedure

Items were loaded onto Palm Pilots (Palm, Inc., Sunnyvale, CA, USA) at the Coordinating Center and distributed to the sites. One question and its responses were programmed per screen. The questionnaires were completed by participants at the schools and downloaded into a central database. We have used Palm Pilots for data collection in other studies [14].

### Criterion Assessment (FV Intake)

Fruit, vegetable and water intakes were assessed using a food frequency questionnaire (FFQ) and up to three 24 hour dietary recalls. Dietary data were collected to document dietary intake in this population, and were used here for validation purposes. FV FFQ intakes were assessed by 10 questions on how many portions of the targeted food were usually eaten at breakfast, lunch, dinner, for a snack after school, at other times, on school days and on non school days, separately (e.g. How many portions of fruit do you usually eat at breakfast on a school day?). The word "portion" was preferred over "serving" because qualitative research among children revealed that a "serving" was how much one puts on their plate, while "portion" connoted some external standard referent for amount. Water intake was assessed with a 10 item FFQ, wherein portion was assessed by number of glasses or bottles. Response categories for all items were "0", "1", "2", and "more than 2."

To obtain a measure of portions, the "more than 2" response was recoded to 2.5. The items were summed with weights of (a) 5, representing the average school week for the school day items and (b) 2, representing the non-school or weekend days. To obtain a daily measure, the summed FFQ score was divided by 7 days.

### Dietary Assessment by 24-hour Recall

Students completed three 24-hour dietary recalls (24hdr). Recalls covered one weekend day and two week days. Trained and certified dietitians obtained the dietary information and recorded it using Nutrition Data System for Research (NDS-R) software (version 4.06_30, 2003, Nutrition Coordinating Center, University of Minnesota, Minneapolis, MN). Phone interviews were conducted by telephone with a food-amounts booklet given to each student that provided dimensional and volume reporting aids for amounts eaten [15-19]. Home telephone interviews were used to minimize missing classroom time for the recalls. One senior dietitian at each site was the designated quality reviewer [20].

### Social Desirability

The 'lie scale' from the Children's Manifest Anxiety Scale [21] consisting of nine dichotomous (yes/no) items was used to ascertain social desirability (SocD). Concurrent validity of the scale has been established [22]. Internal consistency of the scale in this sample was adequate (0.70). The score range was 0-9; higher scores reflected higher socially desirable responses.

### Anthropometry

Anthropometry was conducted in the morning after breakfast. Trained and certified staff collected all measurements using standardized protocols [23] and calibration procedures provided by the study and the equipment manufacturers. The standardization and certification training involved comparing technician trainee measurements to measurements of the same individuals by an accomplished senior technician. Weight was measured twice by one individual using a SECA Alpha 882 scale (SECA Corporation, Hamburg, Germany) and the measurements averaged. Height was measured twice by one individual using a PE-AIM-101 Stadiometer (Perspective Enterprises, Olney, Maryland) and the measurements averaged. BMI z-scores were calculated using the CDC charts [24]. When the weight, or height, measurement difference between the first and second reading were much different (>1 cm for height, >.2 kg for weight), a third measurement was obtained by the same individual, and the two closest values averaged.

### Psychometric Analyses

To decrease bias due to missing data, a priori inclusion criteria of 70% of the items was applied for inclusion in the analyses. Because IRM allows for incomplete data, the item mean value [25] was imputed for classical test theory (CTT) analyses to retain the same set of participants. This method of imputation for the CTT analyses was selected because it was the most conservative, in terms of the mean and in its relationships to other measures (e.g., the IRM analyses). Frequencies and percentages described the demographic characteristics of the sample. Chi-square tests of independence examined differences between participants with and without some SE data.

Initially CTT item analyses were performed to examine item difficulty (item mean and standard deviation), discrimination (corrected item-total correlation, CITC), and scale reliability (Cronbach's alpha). Exploratory factor analyses (EFA) with principal axis factor extraction were then performed to assess the dimensionality of the scales. EFAs were performed on tetrachoric correlations because of the dichotomous nature of the data. EFAs yielded factor loadings for each of the items as well as the percent variance explained by each factor. The EFA is a tool used to demonstrate sufficient unidimensionality whereby subscales may exist [26]. After the assumptions (dimensionality and local independence) necessary for the IRM analyses were verified, Rasch multidimensional IRM analyses were performed using ConQuest [27]. The model contained three dimensions: F, V, W. IRM yielded item parameter difficulty estimates, item infit statistics, Wright maps, and person-separation reliability indices. Infit values can range between zero and infinity; values closer to one indicate agreement between the observed and expected values. Values greater than 1.0 indicate more variation and values less than 1.0 indicate less variation. Ranges from 0.75 to 1.33 indicate good fit for self-reported data [28]. The Wright map visually links the distribution of individuals (indicated by X's on the left side of the Wright map) on the latent SE variable to the distribution of individual item difficulties (represented on the right side by item number). The person-separation reliability index (analogous to Cronbach's alpha [29]), and Cronbach's alpha were assessed. The software did not allow for correction for clustering by school.

IRM used all available data for participants missing ≤ 30% items. IRM incorporates likelihood estimation and expectation-maximization (EM) algorithms to obtain parameter estimates, thereby allowing for missing data and offering greater validity than casewise deletion and simple imputation, assuming the missingness is random [30].

To minimize future participant response burden, item reduction was performed by eliminating items with redundant levels of difficulty. This was accomplished by identifying multiple items within a similar range of difficulty and selecting only one item. IRM was repeated on the reduced sets of items. The complete and reduced sets of items were compared by paired t-tests of the IRM estimated values and by intra-class correlations between self-efficacy estimates. Due to the influence of sample-size on the level of significance, standardized effect sizes (SEF) of the difference between item sets were also provided. The SEF is the difference per unit of the standardized difference. Values of 0.20, 0.50, and 0.80 represent small, medium and large differences, respectively [31].

Construct validity was assessed by correlating (Pearson) the full and abbreviated (reduced) scales with measures of FV and W intake. To control for response bias, all correlations controlled for social desirability.

## Results

**Figure 1:**
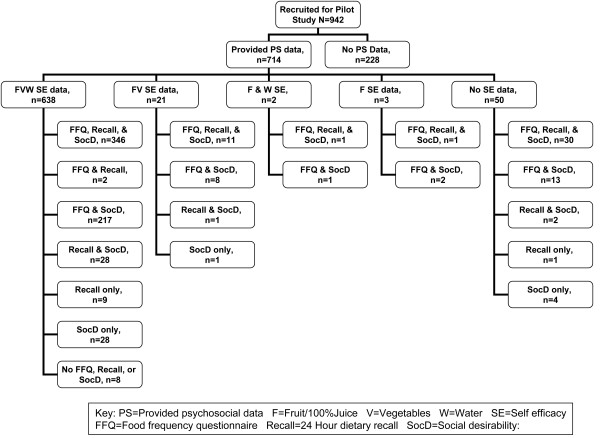
**Flow chart of participant recruitment and availability of complete and incomplete questionnaire and dietary consumption data**.

Although 942 students were recruited to participate in the pilot study, usable SE data were available for only 714 students (see Figure 1). Students were excluded if they (a) provided no psycho-social palm pilot data or had missing/invalid ID numbers (n = 212), (b) had excessive missing data where they did not complete at least 70% of the items on any of the psycho-social scales (n = 16), or (c) did not complete at least 70% of the items within at least one of the FV or W SE questionnaires (n = 53). The final sample was nearly evenly split by gender (Table 2). Approximately one-half of the sample was Hispanic (48.9%) and over one-fourth (27.3%) was Black. Few students (12.0%) had a college graduate head of household. Average student age was 11.3 years (± 0.6) and average BMI%tile was 70.7 (± 28.0).

**Table 2:** Participant Characteristics

Characteristic	Missing data status group	
	
	Missing all SE data	Some or complete SE data with or without social desirability and dietary data
Total^a^	278 (29.5)	664 (70.5)
Gender		
Male	144 (51.8)	325 (48.9)
Female	134 (48.2)	329 (49.5)
Missing^b^	0 (0.0)	10 (1.5)
Race/Ethnicity*		
White	28 (10.1)	96 (14.5)
Black	62 (22.3)	181 (27.3)
Hispanic	159 (57.2)	325 (48.9)
Other	28 (10.1)	50 (7.5)
Missing^b^	1 (0.4)	12 (1.8)
Highest education for head of household n(%)		
HS graduate or less	158 (56.8)	311 (46.8)
Some college or specialized training	70 (25.2)	184 (27.7)
College graduate	39 (14.0)	80 (12.0)
Missing^b^	11 (4.0)	89 (13.4)
Age (in years) n: M (SD)	278: 11.3 (0.6)	654: 11.3 (0.6)
BMI%tile n: M (SD)	275: 73.7 (28.3)	654: 70.7 (28.0)

^a^Total percents are displayed as row percents; remaining percents displayed as column percents

^b^Missing not included in chi-square test of association

*Significant [*X*^2^(3) = 8.76, p = 0.033] between missing data status and race/ethnicity; however, the contingency coefficient (C = 0.10) showed that the association was small. Hispanic participants [OR = 1.7 (1.1, 2.7)] and Other race/ethnicity participants [OR = 1.9 (1.0, 3.6)] were significantly more likely to have missing SE data

The variables were first tested for missing completely at random (MCAR) using Little's likelihood-ratio test [32]. Results indicated that the data were not MCAR (chi-square = 54.22, df = 37, p = 0.015). Bivariate chi-square tests of association between missing data status and demographic characteristics yielded a significant [*X*^2^(3) = 8.76, p = 0.033] association only with race/ethnicity (see Table 2). When MCAR was again tested, after excluding race/ethnicity, the results suggested that data were MCAR (chi-square = 31.32, df = 21, p = 0.068) when not considering race/ethnicity. The bivariate contingency coefficient (C = 0.10) showed this association was small. Hispanic [OR = 1.7 (1.1, 2.7)] and Other race/ethnicity participants [OR = 1.9 (1.0, 3.6)] were significantly more likely to have missing SE data. Because the chi-square is influenced by sample size and the difference was not meaningfully significant, MCAR was tested on 90% of the sample. After randomly selecting 90% of the 942 participants, the 90% of the sample demonstrated MCAR (chi-square = 50.86, df = 37, p = 0.064). Results suggest that the probability of responding to race/ethnicity (and other demographic information) was independent of responding to self-efficacy. As the significant association was more likely due to the sample size and less likely to depend on the strength of the association as evidenced by the contingency association and that the probability of responding to the demographic information was independent of responding to self-efficacy, the data were considered to be MCAR.

The largest sample available was used in each analysis. Listwise deletion, a conservative and less powerful, yet valid method for MCAR, was used where only the 664 students who provided at least some FV and W SE data were included in the psychometric evaluation. A large sub-sample of students (n = 625) who provided social desirability data and at least one measure of dietary consumption were included in the validation phase of the analyses.

The first factor accounted for 38.8% of the variance in the 22 F SE items with a second factor accounting for only an additional 7.5%, indicating, for the purposes of IRM, the scale was sufficiently unidimensional with a single major (or global) dimension. All F SE items had acceptable discriminability (corrected item total correlations) at 0.31 or higher. Cronbach's alpha was 0.84 across all items. IRM of the F SE scale revealed item difficulty estimates ranged from -0.94 (...sure that you can eat 1 portion of fruit for a snack at home at least one time) to 1.11 (...sure that you can eat 1 portion of fruit most times when you eat at a fast food place), and all items were within the fit criteria (Table 1). Person separation reliability (comparable to Cronbach's alpha) was 0.82.

The first factor accounted for 47.1% of the variance in the 14 V SE items, with a second factor accounting for only an additional 9.5% of the variance, indicating a single major dimension scale. All the V SE items had acceptable discriminability at 0.39 or higher. Cronbach's alpha was 0.85 across all items. IRM of the V SE scale revealed that item difficulty estimates ranged from -0.79 (...sure that you can ask someone in your family to serve 2 vegetables for dinner at least one time) to 0.81 (...sure that you can eat 3 portions of vegetables at least 4 days a week, even when you are stressed), and all items met fit criteria (Table 1). Person separation reliability was 0.83.

The first factor accounted for 48.7% of the variance in the seven W SE items (each item loading ≥ 0.51) with a second factor accounting for additional 20.3% of the variance, indicating acceptable unidimensionality. All the W SE items had acceptable discriminability at 0.28 or higher. Cronbach's alpha was 0.70 across all items. IRM of the W SE scale revealed that item difficulty estimates ranged from -0.78 (...sure that you can drink only water whenever you are thirsty for at least one day) to 0.74 (...sure you can drink 6 glasses or bottles of water at least one day), and all items were within fit criteria (Table 1). Person separation reliability was 0.66, which is below acceptable standards (Table 1).

**Figure 2:**
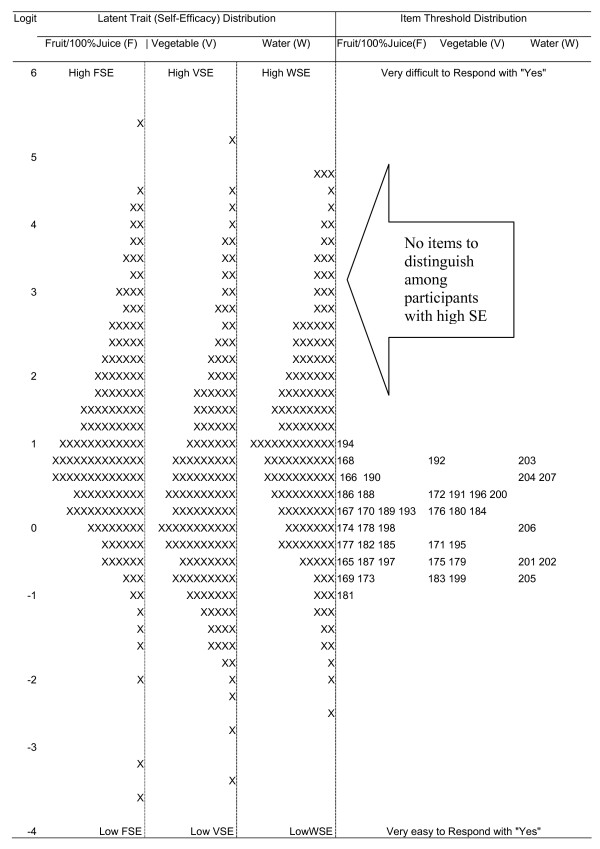
**Wright map of fruit, vegetable, and water self-efficacy latent distribution and item difficulty estimates, with each "x" representing 4.5 cases**.

The Wright maps (Figure 2) revealed that the items in each scale covered only a restricted portion of the distribution covered by participants suggesting inadequate content validity, especially at the more difficult to respond end.

The FV SE scales were highly intercorrelated (r = 0.72) and each was moderately correlated with W SE (F SE with W SE r = 0.50, V SE with W SE r = 0.44).

IRM analyses were repeated with the reduced sets of items (n_fruit _= 10 items; n _vegetables _= 8 items; n _water _= 5 items) with very similar results (not shown). The intra-class correlations between the full and reduced set of FV and W SE items were 0.95, 0.94, and 0.95 for F, V, and W SE scales, respectively (Table 3).

**Table 3:** Correlations among SE and Consumption variables, controlling for social desirability

Partial Correlations	*n*	Fruit	Vegetables	Water
SE_Full _- FFQ, control for SocD	563^a^	0.08	0.20**	0.21**
SE_Full _- 24Hr Recall, control for SocD	374^b^	0.04	0.11*	0.05
SE_Reduced _- FFQ, control for SocD	563^a^	0.08	0.21**	0.20**
SE_Reduced _- 24Hr Recall, control for SocD	374^b^	0.03	0.10*	0.06
FFQ - 24Hr Recall	346^c^	0.17**	0.07	0.14**
SE_Full _- SocD	563	0.03	0.08	0.02
SE_Reduced _- SocD	563	0.02	0.09*	0.03
FFQ - SocD	563	0.20**	0.19**	0.19**
24Hr Recall - SocD	374	-0.04	-0.09	-0.01
Intra-Class Correlations				
SE_Full _- SE_Reduced_	638	0.95	0.94	0.95
24Hr Recall (across days)^d^	432	0.15	0.16	0.42

Abbreviations: Full self-efficacy scale (SE_Full_); reduced self-efficacy scale (SE_Reduced_); social desirability of response (SocD); food frequency questionnaire (FFQ); 24 hour dietary recall (24Hr recall) Significant at p < 0.05 (*) and p < 0.01 (**)

^a ^Participants with fruit, vegetable, and water self efficacy data, social desirability data, and dietary data (FFQ only)

^b ^Participants with fruit, vegetable, and water self efficacy data, social desirability data, and dietary data (24Hr recall only)

^c ^Participants with fruit, vegetable, and water self efficacy data, social desirability data, and dietary data (FFQ and/or 24Hr recall)

^d ^Reliability (ICC) across days was calculated on all participants with or with SE data

Twenty-four hour dietary recalls (24hdr) were obtained on 432 of the children, with most (404, 93.6%) providing three or four days of recall. Single day intraclass correlations (ICC) were low for F (ICC_F _= 0.15), and V (ICC_V _= 0.16) intake, but modest for W (ICC_W _= 0.42) (Table 3). Average (across the three days) ICC were modest for all three types of intake (ICC_F _= 0.35; ICC_V _= 0.37; ICC_W _= 0.68). Mean daily intakes were low with substantial variability for all three intake variables (Table 4).

**Table 4:** Sub-sample means, standard deviations for fruit, vegetable, and water self-efficacy and consumption

	n	SocD X (SD)	Fruit X (SD)	Vegetables X (SD)	Water X (SD)
SE_Full_	591^a^		1.2 (1.7)	0.5 (1.8)	1.1 (1.7)
SE_Reduced_	591^a^		1.1 (1.6)	0.4 (1.9)	1.0 (1.5)
SocD	591^a^	3.6 (2.3)			
FFQ	563^b^		6.7 (2.6)	4.5 (2.5)	7.2 (2.8)
24Hr Recall	374^c^		0.6 (0.7)	1.1 (0.7)	0.9 (1.0)

Abbreviations: Full self-efficacy scale (SE_Full_); reduced self-efficacy scale (SE_Reduced_); social desirability of response (SocD), food frequency questionnaire (FFQ), 24 hour dietary recall (24Hr recall)

^a ^Participants with fruit, vegetable, and water self efficacy data, social desirability data, and dietary data (FFQ and/or 24Hr recall)

^b ^Participants with fruit, vegetable, and water self efficacy data, social desirability data, and dietary data (FFQ only)

^c ^Participants with fruit, vegetable, and water self efficacy data, social desirability data, and dietary data (24Hr recall only)

Internal consistency reliability on the FFQs were 0.83, 0.87, and 0.85 for F, V and W. The mean intakes from FFQ were substantially higher than from 24hdr (Table 4). The FFQ scores were weakly correlated with social desirability, but the 24hdr estimates were not (Table 3).

Only the V SE scale (abbreviated) was significantly correlated with social desirability. Correlations between the SE and intake variables corrected for social desirability, revealed both the long and abbreviated F SE scales were not significantly correlated with F intake. Both the long and abbreviated V SE scales were significantly, but weakly, correlated with V intake by both the FFQ and 24hdr estimates. Both the long and abbreviated W SE scales were significantly, but weakly, related to W intake as estimated by FFQ, but not by 24hdr.

## Discussion

This research attempted to enhance the validity and reliability of existing validated FV SE scales by modifying existing scales to include items that would better assess level (difficulty) of SE and thereby more likely be better distributed across the latent (difficult to respond) variable. The scales were substantially modified, but the distribution across the latent variable was not improved relative to previous versions [10], and the indicators of reliability and validity were not higher. Explanations for lack of expanded distribution may include that 1) the perceived difficulty of the items need even more drastic modification to enhance the distribution; 2) children lack skills to detect difficulty in SE items; or 3) our understanding of the difficulty of FV and W SE is imprecise and we need to add other types of items to manipulate the perceived difficulty. In regard to the latter point, the existing items varied the number of portions and the frequency per week of eating more FV by meal, referred to as situational SE [33]. Items could be restated as specific liked and disliked foods or include method of preparation [4], rather than the generic food category. Other items could be added about confidence in overcoming alternative types of barriers to eating more FV or drinking more water (e.g. motivational, thought process, emotional state, or physical or social impediments [11]) referred to as coping SE [33]. Future research needs to generate additional items and assess which types of item enhance the distributional properties.

The low or lack of correlations between FV SE and corresponding intakes may have been due to 1) poor distribution of items across the latent variable (as shown in figure 1); 2) the weak relationship between SE (of all types) and behavior in young children, regardless of the type of items used [33]; or 3) low intakes of FV and W in this sample with little variance necessary to detect correlations (as shown in Table 4). The lack of significant validity correlations was primarily in regard to fruit. It is possible that SE is not a consideration in regard to consumption of a sweet food item by children. Lower correlations were detected for 24hdr. This appears likely due to a floor effect with very low consumption and low variability. Further research will need to address all these possible explanations.

The reliability and validity coefficients for these expanded scales were low, but comparable to others [10], suggesting these new scales are acceptable measures of these constructs. The low reliability for the water SE scale was likely a function of too few items. The reliability of the criterion variables for tests of validity were similarly low, and likely reduced the obtained correlation coefficients.

An attractive feature of the current analyses was the reduction of respondent burden by selecting one from among redundant items at points along the latent variable to obtain scales with fewer items, but comparable psychometric features. This feature of IRM needs to be more thoroughly tested and explored with measures of a variety of psychosocial variables. A logical progression of these methods would be computer adaptive testing (CAT) of FV and W SE based on IRM modeling, which could even further reduce the numbers of items any individual would have to complete [34,35].

The strengths of this research included a theory based procedure for generating items to enhance the validity of the scales; collecting data from multiple (seven) sites across the US with a reasonably large sample; a narrow age range which minimized differences in cognitive abilities; and application of sophisticated psychometric procedures. The limitations of the research include some data being discarded from the analyses either because of incomplete SE responses (7%) or dietary data (34%). Some on-site observers reported some children provided random responses (but this would have served to diminish psychometric characteristics). It is possible that the FFQ employed was not valid, but similar measures have been validated [36,37]. More days of dietary assessment by 24hdr would have enhanced the reliability of assessment of intake. The IRM psychometric software did not allow for correcting for clustering by school.

In summary, using a theory-based procedure for generating new items to expand the item distribution across a latent variable of FV and W SE among children did not enhance the distributional validity of the new scale, its reliability, or construct validity. Further research, perhaps with items related to SE for overcoming other barriers, is needed to clarify the nature of the problem. Alternatively, this is another example of low correlations of SE with dietary intake, which may simply indicate this is a weak relationship.

## Abbreviations

FV: fruit and vegetable; SE: self efficacy; IRM: Item Response Modeling; W: water; STOPP-T2D: Studies to Treat or Prevent Pediatric Type 2 Diabetes; TX: Texas; CA: California; NC: North Carolina; PA: Pennsylvania; MD: Maryland; FFQ: food frequency questionnaire; 24hdr: 24 hour dietary recall; SocD: social desirability of response; NDS-R: Nutrition Data System for Research; MN: Minnesota; CTT: classical test theory; CITC: corrected item total correlations; EFA: exploratory factor analysis; EM: Expectation Maximization; SEF: standardized effect size; BMI%tile: Body Mass Index percentile; MCAR: missing completely at random; X^2^: chi squared; C: contingency coefficient; OR: odds ratio; ICC: intraclass correlation; CAT: computer adaptive testing; USDA: United States Department of Agriculture; ARS: Agricultural Research Service

## Competing interests

The authors declare that they have no competing interests.

## Authors' contributions

TB was Principal Investigator for this study and wrote a first draft of this manuscript. KW conducted all the statistical analyses and wrote a first draft of the statistical analysis and results sections. CB generated a first draft of the items. JB, KC, and DT participated in item generation and review. AS and all the authors critiqued and edited drafts of this manuscript. All authors read and approved the final version of the manuscript.
